# Rethinking Post-COVID-19 Behavioral Science: Old Questions, New Insights

**DOI:** 10.3390/bs15060831

**Published:** 2025-06-19

**Authors:** Hanvedes Daovisan, Jinpitcha Sathiyamas, Phaktada Choowan, Charin Suwanwong

**Affiliations:** 1 Behavioral Science Research Institute, Srinakharinwirot University, Bangkok 10110, Thailand; 2 Department of Adult Nursing and the Aged, Faculty of Nursing, Thammasat University, Pathum Thani 12120, Thailand

**Keywords:** behavioral science, healthcare behavior, psychological behavior, social behavior, post-COVID-19 pandemic

## Abstract

The COVID-19 pandemic has radically transformed behavioral science research. While many disciplines have been shown increasing attention in the existing literature, behavioral science uniquely revisits old questions to develop new theoretical perspectives for the post-COVID-19 era. Our systematic search of the literature allowed us to map 505 records that met our criteria, found across 102 papers; from these, we chose 11 articles published between 2021 and 2024. The focus of this review is on examining old questions while providing fresh insights into social, psychological, cognitive, healthcare, and human behavior. The findings emphasize the relevance of the TPB, the HBM, SCT, and the COM-B model, which effectively provide new theoretical insights into post-COVID-19 research. This study shows that theory-informed practices have been integrated into behavioral science research since the COVID-19 pandemic. Practical applications depend on these insights, which can inform evidence-based practice of planned behavior in healthcare policy, academic research, and community practice.

## 1. Introduction

Since the WHO declared the end of the COVID-19 pandemic on 5 May 2023, many behavioral science theorists have shown increasing interest in the existing literature (Krpan et al., 2021). Post-COVID-19 research has shown that human behavior has been significantly reshaped, prompting scholars to revisit existing behavioral science theories (Albarracin & Jung, 2021). Previous studies have broadened existing theories to explain the observed increase in behavioral models of health, psychological, and social behavior (Lehmann et al., 2021). Theoretically, increased attention on post-pandemic behavior research can lead to long-term changes in our theoretical models and assumptions (Strickland et al., 2022). The insights gained through this research can support us in comprehending the way in which theory-informed practitioners are revisiting pre-pandemic behavioral models to inform post-COVID-19 research (Pagliaro et al., 2021).

Prior to the COVID-19 pandemic, behavioral science placed great importance on the theory of planned behavior (TPB) (Hagger et al., 2022), which focuses on the relationship between attitudes, subjective norms, and perceived behaviors. However, the pandemic introduced chronic uncertainty, prompting a shift towards theories that account for behavioral models (Wollast et al., 2021). Behavioral models (Albarracin & Jung, 2021) have become more prominent since the COVID-19 pandemic, emphasizing how perceived pathogen threats trigger protective behaviors. This contrast underscores a theoretical evolution in the interplay between behavioral models and science that can be seen in post-COVID-19 research (Chalder, 2024).

According to Lehmann et al. (2021), behavioral models could be useful for social, psychological, cognitive, healthcare, and human behaviors. Post-COVID-19 behavioral science research features behavioral models, such as the TPB, the health belief model (HBM), social cognitive theory (SCT), and the COM-B model (Mosleh et al., 2024). There remains a significant gap in our understanding of the theory-informed practices that have shaped behavioral science in post-COVID-19 research (Almaatouq et al., 2024; Bil et al., 2021; Bonizzato et al., 2022). While the TPB and HBM provide fundamental insights, they often do not integrate the emotional, contextual, or sociopolitical factors that exist in the post-pandemic world. As a result, scholars have requested that behavioral models be updated to better integrate theory-informed practice in post-COVID-19 research (Albarracin & Jung, 2021).

Even though there have been improvements in post-COVID-19 behavioral science theory, there are still significant gaps (Bryan et al., 2021; Dai et al., 2021; Hallsworth, 2023). Current behavioral models emphasize the importance of updated models that include social adjustment in post-COVID-19 research (Shang et al., 2025). In the post-pandemic world, new theoretical answers for theory-informed practice are necessary; the theoretical paradigms of behavioral science have become outdated (Almaatouq et al., 2024; Bryan et al., 2021; Milkman et al., 2021). New questions and insights can be addressed regarding the what, why, where, when, and who of theory-informed practice in post-COVID-19 behavioral science research. Post-COVID-19 behavioral models have expanded their scope, demanding the real-world application of the behavioral science paradigm.

The aim of our systematic mapping review is to categorize theoretical insights in relation to post-COVID-19 behavioral science studies. Our first step involves identifying the behavioral science theories used in post-COVID-19 research in the social, psychological, cognitive, healthcare, and human behavior domains. Second, we map the distribution of theory-informed studies as they relate to behavioral science in post-COVID-19 research. Lastly, we analyze the adaptations made to behavioral models (such as the TPB, the HBM, SCT, and the COM-B model) to address changes in the post-pandemic world. The results visualize the research landscape for practitioners and researchers, allowing them to conduct theoretically informed practice and gain evidence-based insights.

## 2. Materials and Methods

### 2.1. Systematic Mapping Design

This study uses a systematic mapping design (Berger-Tal et al., 2019) to theorize behavioral science in post-COVID-19 research. This systematic mapping design follows the PRISMA framework and applies population, concept, and context (PCC) for the literature search. According to O’Cathain et al. (2013), a systematic mapping design is categorized as a method that summarizes existing theory-informed practices that are related to the research question. It is characterized by its transparent, rigorous, and systematic approach, aiming to construct new theories in an unbiased manner. This design is used to provide a bigger picture for reviewing the literature, enabling us to map the key evidence, concepts, trends, theoretical gaps, and evidence-based research synthesis.

### 2.2. Systematic Mapping Questions

In developing review questions (Bragge et al., 2011), it is necessary to theorize the appropriate route for conducting behavioral science in post-COVID-19 research. The following general question was the driver for this systematic mapping review: to what extent does behavioral science influence post-COVID-19 research? To answer this question, this study addressed five sub-questions by examining the what, why, where, when, and who surrounding behavioral science theories, as presented in Table 1. To answer these five questions, the existing literature was reviewed to identify the research gaps and provide pragmatic knowledge mapping.

### 2.3. Systematic Mapping of the Search Strategy

The mapping search of databases was identified according to the population, concept, and context (PCC) framework for systematic mapping research (Peters et al., 2015). The search string structure was based on five core questions (what, why, where, when, and who), content (identified, described, and synthesized), and outcomes (result outcomes, conceptual research, and theoretical lenses). A systematic search was applied to the following databases, accessed from 1 January 2021 to 31 December 2024: MEDLINE via PubMed, EMBASE via Elsevier, Latin American and Caribbean Health Sciences Literature (LILACS), Cochrane Library Databases, PsyARTICLES, Web of Science, and the Cumulative Index to Nursing and Allied Health Literature (CINAHL). Our systematic mapping review (Tricco et al., 2018) utilized PRISMA guidelines to gather evidence and evaluate the extent of the available literature on a specific topic. Table 2 lists the search strategies for systematic mapping.

**Figure 1:**
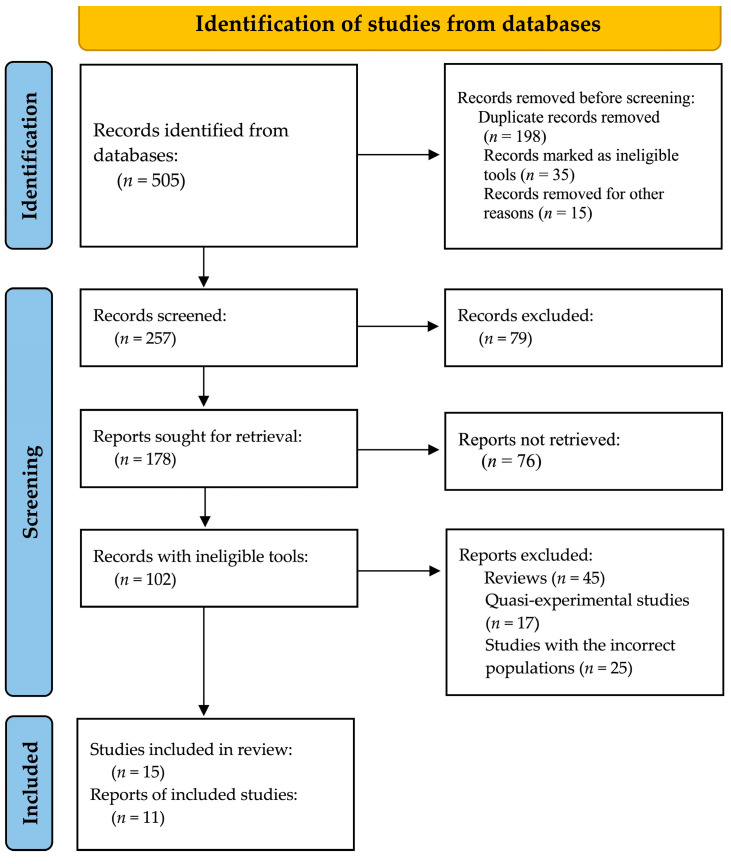
The PRISMA flow diagram.

The PRISMA flow diagram was used to illustrate the selection process of studies for this systematic mapping review using the PCC framework. The first step involved the identification of records through database searches, during which we found 505 records. After removing 198 duplicate records, 35 ineligible tools, and 15 records for other reasons, 257 records remained for screening. Of these, 79 were excluded, leaving 178 reports that were sought for retrieval. The retrieval of 76 reports resulted in the assessment of 102 records as eligible. A total of 87 articles were excluded because they were review studies, quasi-experimental studies, or different population studies. In the final review, there were 15 studies, representing 11 distinct reports that met the selection criteria, as shown in Figure 1.

### 2.4. Systematic Mapping of Eligibility Criteria

This systematic mapping review utilized the PCC framework to structure the eligibility criteria. The population comprised individuals, groups, and organizations that were analyzed in the behavioral science literature. Conceptually, the included studies encompassed behavioral science, including social, psychological, cognitive, healthcare, and human behavior. Contextually, we centered articles on situations that occurred following the COVID-19 pandemic, identifying how new insights were incorporated into the behavioral science paradigm from 2021 to 2024. Only peer-reviewed articles published in English were taken into consideration, while editorials, pre-pandemic studies, and studies that did not address behavioral dimensions were excluded. Table 3 illustrates the eligibility criteria for this systematic mapping review, chosen using the PCC framework.

### 2.5. Systematic Mapping of Data Extraction

Systematic mapping studies extract data to address specific research questions and ensure objectivity and consistency. Our first step was to develop a data extraction form that encompassed publication year, research method, study domain, and key findings. Second, we refined the pilot testing of the form process to eliminate any ambiguities in data interpretation. In the extraction phase, the first author and co-authors worked individually to complete the consensus process and resolve discrepancies. The collaborative approach increased reliability and reduced individual biases. Third, the collected data were then used to create visual representations, such as mapping charts or bubble plots, to identify trends, gaps, and clusters (Peters et al., 2015). Finally, visualization of the findings aided us in building an understanding of how studies are distributed across various categories, highlighting areas for future research.

### 2.6. Systematic Mapping of Quality Assessment

The PCC framework facilitates the use of the existing literature in evaluating quality and identifying trends, research gaps, and the maintenance of scientific rigor (Grimshaw & Russell, 1993). The quality assessment evaluates the primary studies included to ensure that the findings are valid and reliable. This method frequently evaluates the clarity of research questions, the accuracy of data collection methods, and transparency in reporting results (M. Yan et al., 2019). The organization of quality assessment criteria into structured checklists is a common practice that ensures consistent evaluation across studies and ensures that we conduct robust, systematic mapping that can enhance the validity of the conclusions drawn from the body of research. Table 4 presents the quality assessment criteria for mapping the studies.

### 2.7. Systematic Mapping of Data Synthesis

Data analysis in systematic mapping studies involves the categorization of the extracted data to recognize research trends, gaps, and patterns. Our data were analyzed using both quantitative and qualitative techniques to summarize the process of combining the findings of the research studies. Quantitative analysis typically analyzes visualizations, while qualitative synthesis may involve thematic coding (M. Yan et al., 2019). The synthesized data were examined at a summary level to identify the primary research areas and recommend future directions. Table 5 presents the strategies used for synthesizing the data from the systematically mapped studies.

## 3. Results

### 3.1. Preliminary Results

**Figure 2:**
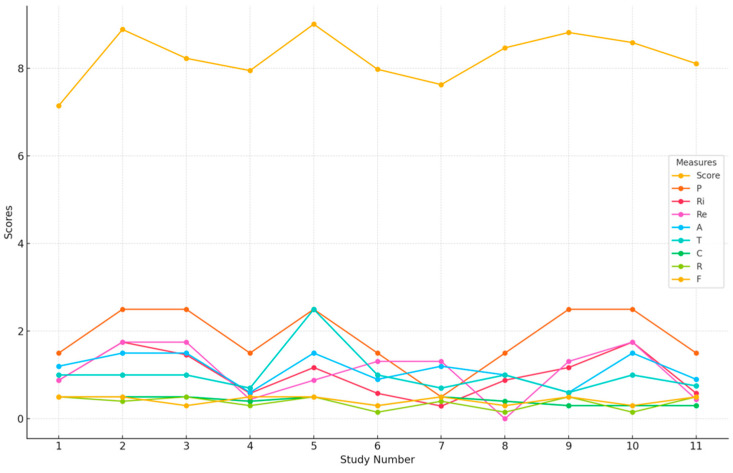
Measuring validity of mapping scores.

**Figure 3:**
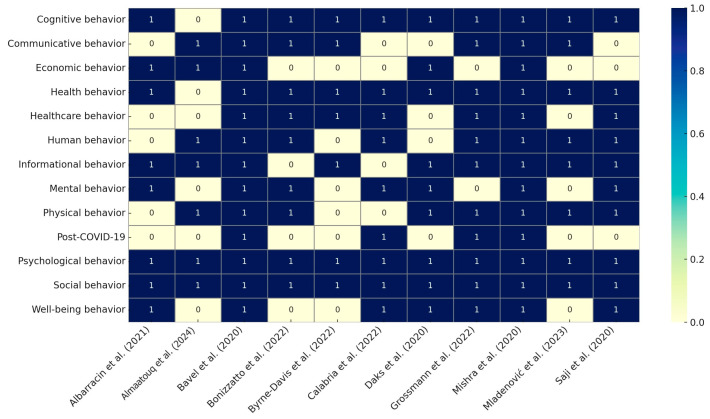
Exact studies in post-COVID-19 behavioral science.

The systematic mapping review approach was chosen to answer old questions for new insights (what, why, where, when, and who) into behavioral science in post-COVID-19 research from 2021 to 2024. From the 11 selected studies, a total of 585 keywords were extracted (250 unique), averaging about 48 keywords per primary study. Of these, 70% of the selected studies focused on new theories of behavioral science in the post-COVID-19 research (Table 6 and Table 7). The behavioral science field validates the ranking of the selected studies according to pertinence, rigor, and relevance. The criteria include publication type, contribution type, research type, and focus trend. Figure 2 shows a summary of the mapping score validity measures used. Figure 3 presents the exact studies chosen from the post-COVID-19 behavioral science literature.

### 3.2. Principal Mapping Results

The results are classified according to each of the answers to the sub-questions (what, why, where, when, and who), as shown in Table 8. The remaining 90.90% of the studies reported the use of a new model and theory, while 27.27% employed an existing evaluation method for behavioral science in post-COVID-19 research. The principal mapping results revealed that the most important questions that were answered in behavioral science in the post-COVID-19 era are those relating to “what”; the answers are presented in the context of the why, the where, the when, and the who in each category, as shown in Table 9.

**Figure 4:**
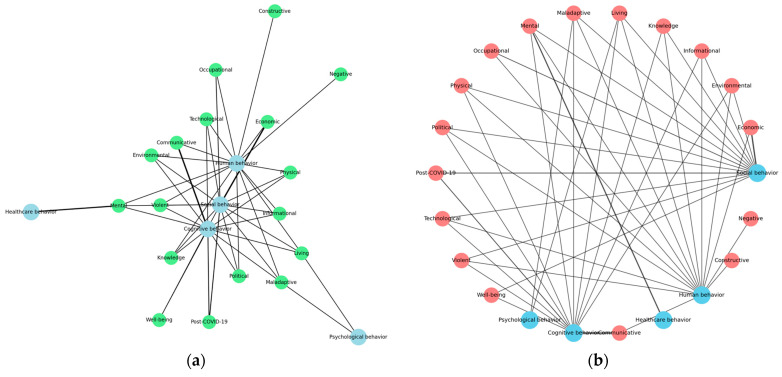
The network analysis of post-COVID-19 behavioral science: (**a**) evidence network; (**b**) theoretical network.

**Figure 5:**
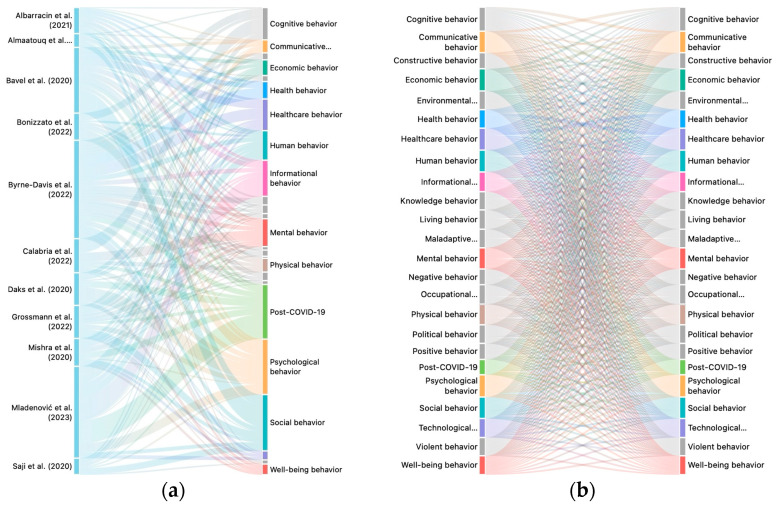
Code co-occurrence mapping study: (**a**) included authors; (**b**) interlinking theories of behavioral science.

A 21-code co-occurrence analysis was conducted to identify the mapping networks of post-COVID-19 behavioral science. Figure 4 shows the networks of the post-COVID-19 behavioral science literature, including the where, the when, and the who that were covered in post-COVID-19 research. The principal mapping results answered the sub-questions, showing that theoretical networks reflect the interdisciplinary nature of behavioral models in a post-pandemic world. The new insights into post-COVID-19 behavioral science serve as hubs, linking the diverse domains of social, psychological, cognitive, healthcare, and human behavior. Post-COVID-19 research has a significant impact on shaping mental, physical, communicative, environmental, and maladaptive behaviors. The co-occurrence mapping study of codes in the post-COVID-19 behavioral science literature is depicted in Figure 5.

### 3.3. Mapping Results

**Figure 6:**
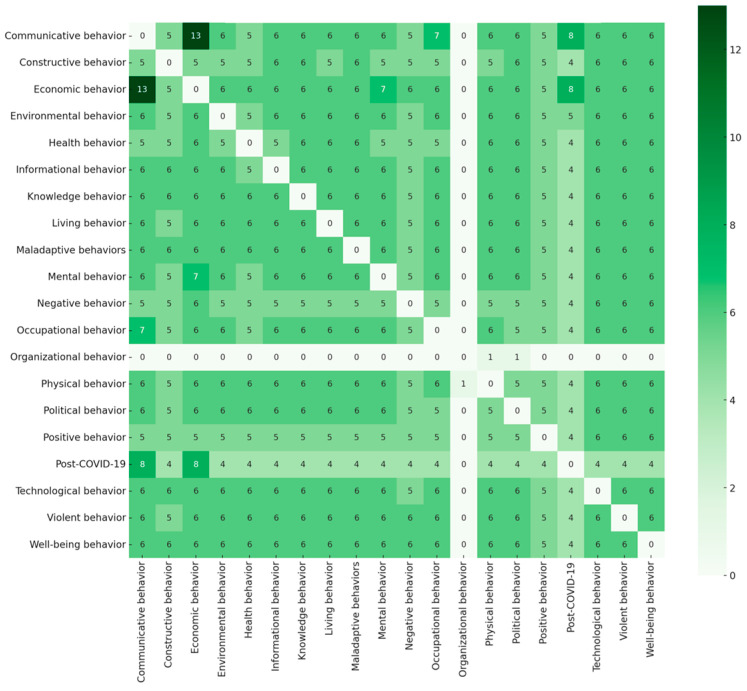
Evidence map of post-COVID-19 behavioral science.

The mapping results of the five core questions revealed that the studies selected from post-COVID-19 research involved new theoretical conceptualizations (90.90%). Around 27.27% of the studies theoretically generated existing, model-based, and/or new evidence for behavioral science. As shown in the first quadrant of Figure 6, category 1 (social behavior) is associated with category 2 (psychological behavior), category 3 (cognitive behavior), category 4 (healthcare behavior), and category 5 (human behavior). The evidence map demonstrates a strong focus on behavioral science in post-COVID-19 research, with significant connections across various behavioral models.

**Figure 7:**
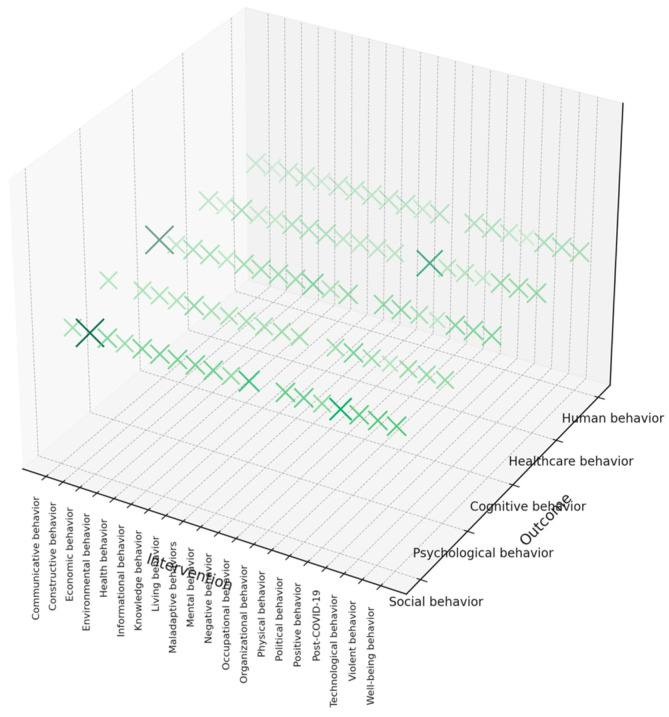
The gap map for post-COVID-19 behavioral science research.

The 3D gap map in Figure 7 shows the connections between various behavioral models (*x*-axis and *y*-axis) in post-COVID-19 behavioral science. Each *x*- and *y*-axis mark denotes the presence and density of research intersections. The visualization reveals that although there are many themes (human, cognitive, and healthcare behavior), significant gaps still remain in various areas. The well-covered areas of mental, economic, and healthcare behavior have been extensively studied in post-COVID-19 research. The moderate exploration surrounding technological, cognitive, and informational behaviors suggests that attention is growing in these areas, but it is still developing. The underrepresentation of occupational, constructive, environmental, and well-being behavior research indicates that these areas should be explored in the future.

**Figure 8:**
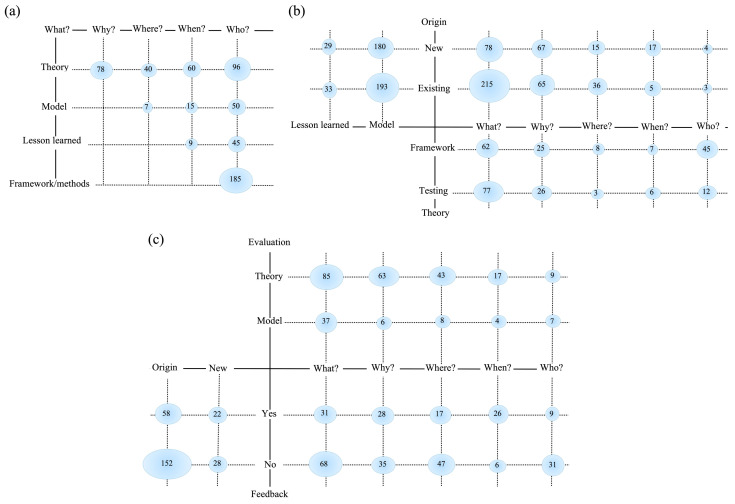
The core questions for post-COVID-19 behavioral science are: (**a**) the focus of mapping; (**b**) the nature of contributions; and (**c**) the structure of feedback.

Figure 8 presents the outputs of the combination of the five core questions (what, why, where, when, and who) in a black-box model. By analyzing the mapping results shown in the three bubble charts in Figure 8a–c, the following observations can be made. Looking at the pertinence facet in Figure 8a, covering systematic mapping focus, the remaining 11 selected studies (45.45%) include philosophical papers, solution proposals (36.36%), and evaluation research framework/methods, accounting for lessons learned (9.09%). Figure 8b depicts the systematic map of contribution, pertinence, and research type, highlighting the focus on new, existing, standard, and ad hoc approaches. Figure 8c illustrates the mapping results obtained from feedback on the type of evaluation methods. The evaluation approaches, theories, models, and feedback methods used in the post-COVID-19 behavioral science.

**Figure 9:**
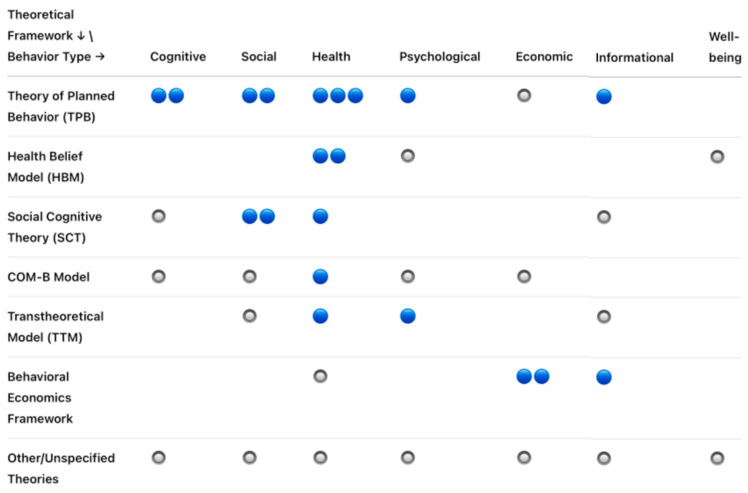
The conceptual layout of post-COVID-19 behavioral science.

**Figure 10:**
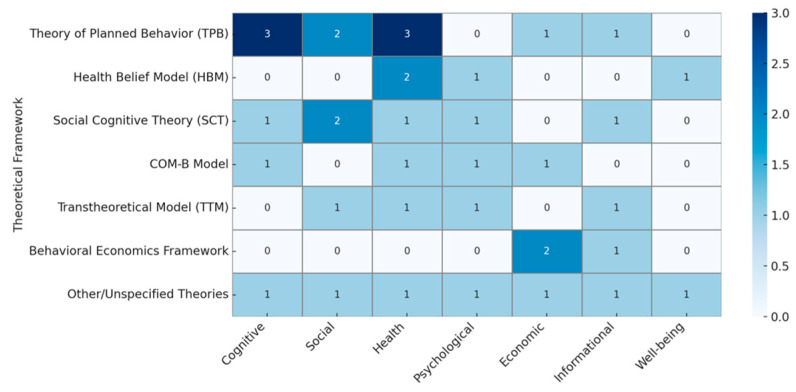
The theoretical layout of post-COVID-19 behavioral science.

An evidence map shows the research that exists on a broad topic in behavioral science related to post-COVID-19 research. The key dimensions of this mapping review, i.e., theoretical frameworks, outcomes, and study quality, are depicted in Figure 9. The TPB has been extensively studied in health and social behaviors, while the HBM is primarily focused on health and psychological behaviors, and SCT enables the intersection of social and cognitive behavior. The COM-B provides a wide-ranging coverage that includes economic behavior in theoretical frameworks. The structure of the evidence maps (as can be seen in Figure 10) is as follows: the *x*-axis covers post-COVID-19 behavioral science (cognitive, social, health, psychological, economic, information, and well-being behaviors), and the *y*-axis covers the relevant theoretical frameworks (TPB, HBM, and SCT).

**Figure 11:**
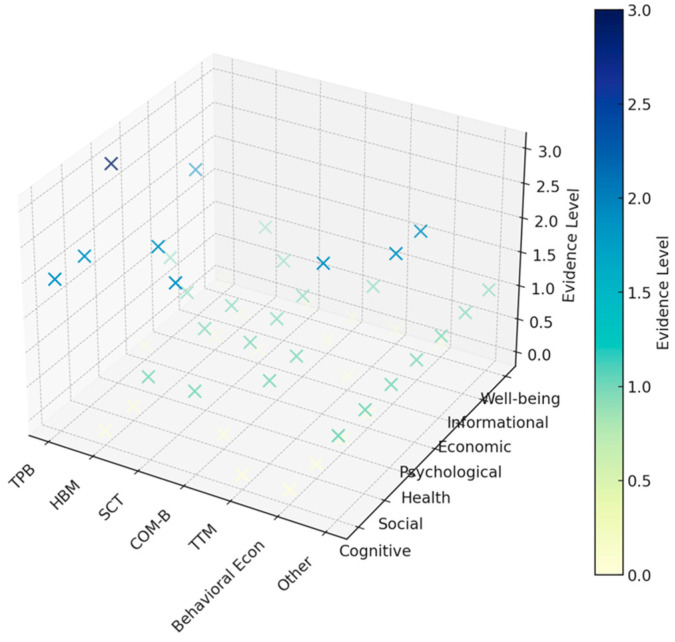
Combining theoretical models and evidence for post-COVID-19 behavioral science.

The 3D map in Figure 11 illustrates the relationships between theoretical models (*x*-axis) and various behavioral models (*y*-axis) on the evidence map (*z*-axis) in post-COVID-19 behavioral science. The strongest theoretical models include the TPB, the HBM, and SCT—indicating a solid theoretical foundation in the field of behavioral science in social, psychological, healthcare, and human behaviors. Models of economic, informational, and well-being behaviors are applied consistently, revealing critical gaps in theoretical integration. Economic behavior and the COM-B are underutilized, despite their relevance to behavior models for post-COVID-19 behavioral science research. However, newer and context-relevant models of the COM-B are still underapplied, and there is limited theoretical grounding for them in post-COVID-19 research.

## 4. Discussion

### 4.1. RQ1—What?

Within category 1, new insights on social behavior in post-COVID-19 research were found to be related to three behaviors: environment, living, and technological behaviors (Bonizzato et al., 2022; Mladenović et al., 2023). The available theoretical explanations of social behavior are applicable to economics, sociology, psychology, psychiatry, medicine, and physiology (Hayes et al., 2012); moreover, we theorized a new behavioral science paradigm for post-COVID-19 research (Bonizzato et al., 2022). Some studies have a typical new behavioral science lens (Daks et al., 2020); however, we reintegrate social behavior insights into post-COVID-19 research.

The new key concepts of social behavior in post-COVID-19 research involve behavioral adherence, social cohesion, prosocial behavior, and collective behavior (Lehmann et al., 2021; Mladenović et al., 2023; Saji et al., 2020). Previous studies have revealed the importance of social behavior in post-COVID-19 research, which is associated with insights into psychological support, social norms, the HBM, the TPB, and community engagement (Albarracin & Jung, 2021). The post-COVID-19 literature provides substantial insights into what “social behavior” might constitute, being a transdisciplinary topic that typically encompasses a behavioral model (Almaatouq et al., 2024; Bavel et al., 2020; Bonizzato et al., 2022; Mladenović et al., 2023; Olapegba et al., 2022).

### 4.2. RQ2—Why?

Within category 2, the new understandings of psychological behavior gained from post-COVID-19 research cover maladaptive, well-being, and personal behaviors (Bavel et al., 2020; Calabria et al., 2022; Mishra et al., 2020; Mladenović et al., 2023). According to Albarracin and Jung (2021), Lehmann et al. (2021), and Ovretveit et al. (2021), psychological behavior is associated with resilience, motivation, behavioral fatigue, and psychological adaptation. Recent studies have provided new theoretical insights into the why of psychological behavior in post-COVID-19 research using the TPB, the HBM, SCT, and the COM-B (Bonizzato et al., 2022; Lehmann et al., 2021; Teichman & Underhill, 2021). Previous studies suggest that insights into psychological behavior are associated with self-efficacy, intentions, behavioral inertia, and post-traumatic growth in post-COVID-19 research (Mishra et al., 2020; Mladenović et al., 2023).

### 4.3. RQ3—Where?

Within category 3, the new insights into cognitive behavior revealed in post-COVID-19 research are related to positive, negative, and violent behaviors (Almaatouq et al., 2024; Byrne-Davis et al., 2022; Mishra et al., 2020; Mladenović et al., 2023). In the context of post-COVID-19 research, studies have examined cognitive dissonance, cognitive flexibility, cognitive load, and decision making (Matiza & Kruger, 2021; Sahu & Rao, 2023). Albarracin and Jung (2021), Chater and Loewenstein (2023), and Lehmann et al. (2021) stated that cognitive behavior, involving cognition, has an impact on behavior, cognitive distortions, and maladaptive thinking patterns. Similarly, Bryan et al. (2021) found that cognitive behavior is a good way to conceptualize thinking patterns, the HBM, the TPB, memory retention, and mental flexibility in post-COVID-19 research.

### 4.4. RQ4—When?

Within category 4, the key insights into healthcare behavior relate to physical, mental, and occupational behaviors in post-COVID-19 research. Our current understanding of the when in healthcare behavior, according to post-COVID-19 research, is associated with preventive behavior, health literacy, and health-related risk perception (Baumann & Cabassa, 2020; Miller et al., 2021). Previous studies have reconceptualized new insights into healthcare behavior that encourage service providers to engage in post-COVID-19 research (Lee & Lee, 2021). It is possible that the HBM, the TPB, and the COM-B used in post-COVID-19 research may be harmful when healthcare behavior emerges (Bosnjak et al., 2020; Chalder, 2024; Hallsworth, 2023; Mosleh et al., 2024; Saji et al., 2020; Wollast et al., 2021).

### 4.5. RQ5—Who?

Within category 5, we found that the new insights into human behavior relate to communicative, knowledge, economic, and political behaviors in post-COVID-19 research (Byrne-Davis et al., 2022; Daks et al., 2020; Mladenović et al., 2023). New research on human behavior since COVID-19 has been linked to changing behaviors, developing habits, and fostering social responsibility (Albarracin & Jung, 2021; Lehmann et al., 2021). Post-COVID-19 research has involved the clarification of new insights into human behaviors based on SCT, the TPB, and transtheoretical models (Bil et al., 2021; Bryan et al., 2021; Mladenović et al., 2023). Our focus is on who contributed to research into the interactions between cognitive processes, emotions, social influences, environmental conditions, and individual motivations in post-COVID-19 research.

### 4.6. Theoretical Contributions

This systematic mapping review provides some theoretical contributions. First, the results offer theoretical insights into new versions of existing behavioral science theories (Glanz & Bishop, 2010), such as the TPB (Zhao & Gao, 2022), the HBM (Yuen et al., 2021), SCT (Hagger & Hamilton, 2022), and the COM-B (J. Yan et al., 2024), in post-COVID-19 research. Our findings have unambiguously filled the theoretical gap in post-COVID-19 behavioral science (Ruggeri et al., 2024; Shang et al., 2025); the new versions of existing theoretical behavioral models focus on the current state of social, psychological, cognitive, healthcare, and human behaviors. Theoretical contributions to social behavior following COVID-19 are based on the established SCT instead of the TPB, with both providing distinct yet complementary insights (Lucarelli et al., 2020). The HBM provides insights into psychological behavior, focusing on self-efficacy, cognitive dissonance, and perceived susceptibility (Sarwar et al., 2023). Meanwhile, the COM-B synthesizes behavioral, psychological, and contextual factors, comprehensively accounting for healthcare behavior in post-COVID-19 research (Tesema et al., 2021).

### 4.7. Practical Implications

This systematic mapping review provides some important insights into the practical implications of our findings. First, our findings provide new practical insights into the “what?” of the literature, identifying the following social behaviors that are centered in post-COVID-19 research: behavioral adherence, social cohesion, prosocial behavior, and social support seeking. Second, our new practical understandings of the “why?” surrounding psychological behavior in post-COVID-19 research relate to resilience, well-being, mental health outcomes, and personal behaviors. Third, our extensive insights into the “where?” of the literature show that cognitive behavior is central: positive, negative, and mental flexibility shape practical behavioral science. Fourth, we found that healthcare behavior insights can be encouraged through physical, mental, and health literacy, in addition to occupational behaviors, in post-COVID-19 research. Our analysis concluded that human behavior insights may be influenced by communicative, knowledge-based, economic, and political behaviors; this insight is advantageous for those who engage in post-COVID-19 research. These practical implications highlight the potential for academic, organizational, institutional, and healthcare policies to be implemented, which could aid post-COVID-19 behavioral science research.

### 4.8. Limitations and Future Research

This systematic mapping review of the post-COVID-19 behavioral science literature has some limitations that warrant consideration for the benefit of future research. First, a key limitation in post-COVID-19 research from 2021 to 2024 is the limited availability and scope of data that are specifically grounded in behavioral science. Second, the included studies lack longitudinal data, limiting potential insights into how behavioral science has changed in post-COVID-19 research policies. Third, the dominance of studies from high-income countries results in a geographic bias, which reduces the generalizability of the findings to low- and middle-income contexts. Fourth, while theoretical models, such as the TPB, the HBM, SCT, and the COM-B, are reconceptualized, there is limited integration, hindering comprehensive behavioral insights. Fifth, there are inconsistencies in the terminology of behavioral science in post-COVID-19 research across the analyzed studies, which used meta-analyses of social, psychological, cognitive, healthcare, and human behavior. To address these shortcomings, future research should concentrate on standardizing methodology, integrating theory, using population samples, and utilizing mixed-method approaches to behavioral science in post-COVID-19 research.

## 5. Concluding Remarks

This systematic mapping review of behavioral science in post-COVID-19 research addresses old questions as new insights across five key dimensions: what, why, where, when, and who. Our theoretical mapping model brings new insights to old questions related to social, psychological, cognitive, healthcare, and human behaviors in the context of post-COVID-19 behavioral science. The findings highlight the importance of the new behavioral models (the TPB, the HBM, SCT, and the COM-B), which offer crucial frameworks for post-COVID-19 research. A theory-driven method is essential for comprehending the full impact of behavioral science insights on the behavioral models that are used in post-COVID-19 research. The findings underscore the importance of contextualizing evidence-based models and theory-informed practices in answering the questions of what, why, where, when, and who in a post-pandemic world.

## Figures and Tables

**Table 1:** Formulation of core and sub-questions.

Core Questions	Sub-Questions
1. What?	What behavioral science can be used to explain social behavior?
2. Why?	Why is behavioral science involved in psychological behavior?
3. Where?	Where has behavioral science successfully implemented cognitive behavior?
4. When?	When did behavioral science shift healthcare behavior?
5. Who?	Who had the most impact from behavioral science in evaluating human behavior?

**Table 2:** Key term search strategies.

Core Concept	Terms
Behavioral science	behavioral science*; behavioral paradigm*; behavioral theory*; behavioral concept*; behavioral principle*; behavioral method*; behavioral practice*
Post-COVID-19	post-COVID-19 pandemic*; post-COVID-19 era*; post-COVID-19 world*

**Table 3:** Eligibility criteria using the PCC framework.

PCC Element	Inclusion Criteria	Exclusion Criteria
Population	Research that involves the target population (individuals, groups, and organizations)	Studies involving unrelated populations (children; public, if not relevant)
Concept	Focus on the defined topic/phenomenon in behavioral science (social, psychological, cognitive, healthcare, and human behavior)	Studies not addressing the defined concept (physical health interventions)
Context	Conducted in the specified setting (post-COVID-19 pandemic from 2021 to 2024)	Studies outside the context (pre-COVID-19 and during COVID-19 in unrelated geographic/temporal contexts)
Criteria type	Published in English within a specified timeframe with peer review	Non-English studies, editorials, opinion pieces, and non-peer-reviewed works are examples of non-English study types

**Table 4:** Quality assessment criteria.

Criterion	Description
The clarity of research questions	Determines whether the study clearly defines its objectives
Appropriateness of the methodology	Determines whether the chosen methods suit the research goal
Completion of data reporting	Determines whether all data and results are fully disclosed
Relevance to the research subject	Determines whether the study aligns with the mapping focus

**Table 5:** Classification of data synthesis strategies.

Strategy	Description	What	Why	Where	When	Who
Frequency analysis	Counting occurrences of specific attributes	✓	✓	✓	✓	✓
Thematic coding	Grouping studies based on recurring themes or concepts	✓	✓	✓	✓	✓
Clustering techniques	Organizing studies into clusters based on similarities	✓	✓	✓	✓	✓
Visualization	Graphical representation of data distribution	✓	✓	✓	✓	✓

**Table 6:** Mapping overview.

Author (Year)	Study Type	Contribution	Focus	Pertinence
1. Albarracin and Jung (2021)	Philosophical papers	Model	Psychological behavior in post-COVID-19 research	Full
2. Almaatouq et al. (2024)	Philosophical papers	Theory	Human behavior in post-COVID-19 research	Full
3. Bavel et al. (2020)	Solution proposal	Model	Social behavior in post-COVID-19 research	Full
4. Bonizzato et al. (2022)	Solution proposal	Theory	Cognitive and psychological behavior in post-COVID-19 research	Full
5. Byrne-Davis et al. (2022)	Philosophical papers	Model	Healthcare behavior in post-COVID-19 research	Full
6. Calabria et al. (2022)	Philosophical papers	Model	Cognitive behavior in post-COVID-19 research	Full
7. Daks et al. (2020)	Philosophical papers	Model	Contextual behavior in post-COVID-19 research	Full
8. Grossmann et al. (2022)	Philosophical papers	Theory	Societal behavior in post-COVID-19 research	Full
9. Mishra et al. (2020)	Solution proposal	Lesson learned	Economic behavior in post-COVID-19 research	Full
10. Mladenović et al. (2023)	Philosophical papers	Theory	Emotional behavior in post-COVID-19 research	Full
11. Saji et al. (2020)	Evaluation research	Framework/methods	Social behavior in post-COVID-19 research	Partial

**Table 7:** Selected mapping study.

Ref.	Origin	Type(s)	Stage(s)	Feedback	Validated
1	New	Theorizing	Design	Yes	Experiment
2	New	Integrative experiments	Design	Yes	Testing theories
3	Existing	Theoretical framework	Design	No	Conceptualizing
4	New	Observation	Testing	Yes	ANOVA
5	New	COREQ guidance	Design	Yes	Rigor
6	New	Partial correlations	Testing	Yes	Regression
7	Existing	Predicting	Testing	No	Model
8	New	Estimating	Design	Yes	Multiple analysis
9	New	Theoretical framework	Design	No	–
10	New	Hypothesizing	Design	Yes	Moderation analysis
11	Existing	Conceptual framework	Design	No	Cross-sectional survey

**Table 8:** Results of the principal mapping review.

Research Sub-Question	Possible Answer	Results	
		#Studies	%
1. What?	New model	9	90.90
2. Why?	Existing framework	3	27.27
3. Where?	New evidence	3	27.27
4. When?	New theory	9	90.90
5. Who?	New implementer	3	27.27

**Table 9:** Summary of answers to the five core questions and recurrent themes.

Core Question	Exemplary Answers Derived from the Extant Literature
1. What?	To discover what social behavior in post-COVID-19 research relates to environmental, living, and technological behaviors (Bonizzato et al., 2022; Byrne-Davis et al., 2022; Daks et al., 2020).
2. Why?	To discover why psychological behavior in post-COVID-19 research encourages maladaptive, well-being-related, and personal behaviors (Mishra et al., 2020; Mladenović et al., 2023).
3. Where?	To discover where cognitive behavior in post-COVID-19 research is dependent on mental, positive, negative, and violent behavior (Almaatouq et al., 2024; Bavel et al., 2020).
4. When?	To determine when healthcare behavior in post-COVID-19 research became associated with physical, mental, and occupational behaviors (Albarracin & Jung, 2021; Bonizzato et al., 2022; Grossmann et al., 2022; Saji et al., 2020).
5. Who?	To discover who is most frequently involved in human behavior in post-COVID-19 research associated with communicative, knowledge, economic, and political behaviors (Bonizzato et al., 2022; Calabria et al., 2022; Daks et al., 2020).

## Data Availability

No new data were created or analyzed in this study.
